# School‐Related Stressors, Pain Outcome Measures and Extremely Low Health‐Related Quality of Life in a Representative Sample of Hungarian Adolescents With Functional Abdominal Pain Disorders

**DOI:** 10.1155/ijpe/9964179

**Published:** 2026-05-27

**Authors:** János Major, Adrienn Vargay, Szilvia Ádám

**Affiliations:** ^1^ Institute of Behavioural Sciences, Semmelweis University, Budapest, Hungary, petho.hu; ^2^ Paediatric Pain Centre, HRC Bethesda Children′s Hospital, Budapest, Hungary; ^3^ Institute of Psychology, ELTE Eötvös Loránd University, Budapest, Hungary, elte.hu; ^4^ Health Services Management Training Centre, Semmelweis University, Budapest, Hungary, petho.hu

**Keywords:** abdominal pain, adolescent health, chronic pain, epidemiology, irritable bowel syndrome

## Abstract

**Background:**

Despite the growing body of evidence on paediatric chronic pain, there is a paucity of studies focusing on school stressors, internationally recommended core outcome measures for chronic pain and chronic disabling pain in adolescents with functional abdominal pain disorders (FAPDs).

**Methods:**

We conducted a cross‐sectional, nationwide study in a representative sample of 657 adolescents (51.1% girls, median age 15.0 [IQR 13.0–17.0] years) including 62 adolescents with FAPDs. We used study‐specific questionnaires to explore school stressors, core outcome measures of pain and other somatic symptoms. We deployed multiple regression analyses to identify predictors of extremely low health‐related quality of life (HRQOL).

**Results:**

Amongst adolescents with FAPDs, school stressors and frequent harassment were significantly more prevalent; extraintestinal pain was reported by 54.8%, and 11.3% have reported continuous or daily pain. Significantly lower HRQOL, a higher number of missed school days and more frequent use of pain medication were identified. In the FAPD group, 14.5% (*N* = 9) adolescents reported extremely low HRQOL, which was associated with verbal harassment, multisite pain and higher frequency of pain on multivariate analysis.

**Conclusions:**

Our results suggest that a portion of adolescents with FAPD may suffer from chronic disabling pain with extremely low HRQOL. These results may guide more accurate diagnostics of FAPDs.

## 1. Introduction

Functional abdominal pain disorders (FAPDs) are amongst the most common manifestations of chronic pain in childhood and adolescence, affecting approximately 13.5% of children worldwide [1]. These disorders are associated with impaired daily functioning, reduced health‐related quality of life (HRQOL), increased healthcare utilisation, medication overuse, psychiatric comorbidity and elevated risk of persistent symptoms into adulthood [2–6].

Because school constitutes a major part of adolescents′ everyday environment, school‐related stressors represent an important but still understudied psychosocial context for FAPDs. Available evidence suggests that school stress, peer problems and harassment may contribute to both the development and maintenance of abdominal pain. A landmark study by Hjern et al. demonstrated that school harassment and psychosocial school stressors were significant predictors of chronic abdominal pain [7]. Similar associations have been reported in population‐based studies from different regions, linking emotional stress, school difficulties and peer problems to functional gastrointestinal symptoms [2, 8, 9].

However, not all adolescents with FAPDs experience clinically relevant disability. Population‐based data indicate that whilst many adolescents meet diagnostic criteria, only a subset develops severely disabling pain characterised by persistent symptoms, markedly reduced quality of life, school absence and frequent healthcare use [10]. Identifying this high‐risk subgroup is clinically crucial, as these patients are most likely to require highly specialised, multimodal, interdisciplinary pain management [5, 11].

Despite this, paediatric gastroenterology literature has only limited data on how to identify adolescents with FAPDs who are at risk for chronic disabling pain. Previous studies have examined quality of life, psychological functioning and healthcare utilisation in FAPDs, but few have applied internationally recommended outcome frameworks [4, 12–14]. In particular, most studies did not follow the Core Outcome Domains and Measures for Pediatric Acute and Chronic/Recurrent Pain Clinical Trials (PedIMMPACT) recommendations, which emphasise assessment of pain, physical and psychosocial functioning, role functioning, sleep and economic impact [15]. Without these standardised outcome domains, it remains difficult to compare findings across studies or to reliably identify clinically severe cases.

Furthermore, data from Central and Eastern Europe are scarce. In Hungary, only one population‐based study has previously examined FAPDs, reporting a point prevalence of 11.9% [16]. However, no study has yet explored how school stressors, pain characteristics and functional consequences interact in this population, nor which factors distinguish adolescents with mild FAPDs from those with disabling disease.

Building on our previous epidemiological work, the present study addressed these gaps by applying a comprehensive, PedIMMPACT‐aligned assessment in a representative national sample of Hungarian adolescents [16]. Specifically, amongst adolescents with FAPDs, we aimed to

1. Characterise school‐related stressors and peer harassment,
2. Describe pain severity, multisite pain and associated somatic symptoms,
3. Assess pain‐related disability and functional consequences (HRQOL, school absenteeism, sleep problems, healthcare utilisation and analgesic usage),
4. Identify a subgroup with extremely low HRQOL,
5. Determine which pain‐ and school‐related factors predict membership in this high‐risk subgroup.

We hypothesised that, compared with adolescents without FAPDs,

1. School stressors and peer harassment would be more prevalent amongst adolescents with FAPDs;
2. Adolescents with FAPDs would report multisite pain, more severe pain characteristics and a higher burden of additional somatic symptoms;
3. Adolescents with FAPDs would show poorer HRQOL and more adverse functional consequences (e.g. school absence and analgesic usage);
4. A clinically meaningful subgroup of adolescents with FAPDs would exhibit extremely low HRQOL (≤ −2 SD);
5. Extremely low HRQOL would be associated with greater pain severity and higher exposure to school‐related stressors and harassment.

## 2. Materials and Methods

### 2.1. Sample

We conducted a national cross‐sectional study in April 2016. Our sample consisted of adolescents between the ages of 11 and 18 years, as well as their parents from 22 schools spanning all eight regions in Hungary. Students were randomly selected every fifth pupil from each class using the Hungarian Statistical Yearbook of Education 2013/2014, to ensure that the sample had matching sociodemographic characteristics to those of the normative population, as described earlier. The minimal sample size (*N* = 602) was determined based on data from previous epidemiological studies (e.g. total prevalence of 10%, 95% confidence interval, degree of absolute precision of 5% and 20% attrition) [16]. The study population was representative of every school grade, sex and region in Hungary. We attempted to collect data from a variety of socioeconomic groups within the population by recruiting schools from less affluent areas. Previous research deemed this to be of particular significance in the context of chronic pain [17].

Of the 657 questionnaires distributed to participating schools, 527 were returned, yielding a response rate of 80.2%. Students with parental consent completed the anonymous questionnaires during class time within 2 weeks, after which teaching staff collected and returned the forms to the research team. Parents were additionally asked to complete the same questionnaire (data not shown). Five questionnaires were excluded due to missing or insufficient data (e.g. missing information on sex or inadequate information to establish an FAPD diagnosis). Consequently, 522 questionnaires (79.5%) were included in the final analyses.

The final sample comprised 51.1% girls (*N* = 267; median age = 15.0 years, IQR = 13.0–17.0). Based on the Questionnaire for Paediatric Gastrointestinal Symptoms Rome III Edition (QPGS‐III), 62 adolescents met the criteria for an FAPD, as reported previously [16]. Rome III criteria were applied because the study was initiated prior to the publication of the Rome IV criteria. Adolescents who did not meet the criteria for an FAPD served as controls (*N* = 460), as described in detail elsewhere [16]. No medical examination was performed as part of the study.

### 2.2. Setting

Printed questionnaires and informed consent forms were mailed to each school involved in the study. Teachers asked the students whose parents consented to the study to complete the anonymous questionnaires during school time within 2 weeks of receipt of the questionnaires. The test battery comprised approximately 100 questions (variations based on the QPGS‐III, which tailors questions to specific symptoms), with an average completion time of 45 min for each assessment. Completed questionnaires were collected by teachers and mailed back to our research group.

### 2.3. Main Outcome Measures

#### 2.3.1. School Stressors

School‐related stressors and experiences of harassment were assessed using the questionnaire developed by Hjern et al. [7]. In the present study, we have used 10 items from this instrument. The questionnaire comprised three content domains: perceived school workload and pace (one item), school environment stressors (four binary items assessing lack of calmness in the classroom [no peace and quiet in the classroom] and dining hall [no peace and quiet in lunchroom], problematic teacher–student relationships [treated unfairly by teacher] and insufficient time for lunch [too little time for lunch]) and peer harassment experiences (four items assessing accusation, exclusion, verbal aggression and physical aggression). In the original instrument by Hjern et al., peer harassment is assessed using four distinct items: accusation, exclusion, verbal aggression and physical aggression [7]. The item ‘accusation’ referred to experiences of being blamed, falsely accused or made a scapegoat by peers, representing a form of relational or social victimisation rather than overt verbal or physical aggression. ‘Exclusion’ referred to experiences of being deliberately left out of social activities or peer groups, reflecting social rejection and isolation. ‘Verbal aggression’ referred to being subjected to hostile or hurtful spoken interactions, such as teasing, mocking or being called names by peers. ‘Physical aggression’ referred to direct bodily acts of hostility, such as being hit, pushed or physically intimidated by peers. Harassment items were rated on a 5‐point Likert scale ranging from 1 (*daily*) to 5 (*never*), whilst school environment items were scored dichotomously (yes/no). Items were analysed individually rather than as a composite scale, and therefore, no total score or unified score range was calculated.

Harassment frequency was evaluated both as an ordinal variable and as a derived categorical indicator, defining ‘frequent harassment’ as exposure occurring at least on a weekly basis, in accordance with the original publication. No established clinical cutoffs exist for this instrument; higher frequency ratings indicate greater exposure to school‐related stressors or harassment. As the questionnaire consists of heterogeneous items capturing distinct aspects of school stress and peer victimisation, internal consistency indices (Cronbach′s *α*) were not reported in the original validation study and were not calculated in the present sample. The questionnaire was translated and culturally adapted into Hungarian for the purposes of this study; however, no prior formal psychometric validation in Hungarian samples has been published. The analytic approach followed the methodology applied in the original study by Hjern et al. [7].

#### 2.3.2. Pain Characteristics

Pain intensity was assessed using the 11‐point numeric rating scale (NRS), a single‐item self‐report measure ranging from 0 (*no pain at all*) to 10 (*worst imaginable pain*) [18]. The NRS yields a continuous score between 0 and 10, with higher scores indicating greater pain intensity. As a single‐item instrument, the NRS does not include subscales, and internal consistency indices (Cronbach′s *α*) are not applicable. No universally established clinical cutoff values exist; scores are interpreted dimensionally, with higher values reflecting more severe pain. The NRS has been widely validated and is recommended for use in paediatric and adolescent pain research, including international and Hungarian clinical contexts. In the context of acute pain, pain intensity constitutes a pivotal measurement. However, in the case of chronic pain, the implementation of additional metrics is imperative to facilitate a more comprehensive evaluation of the severity of the pain [18].

The prevalence and additional characteristics of pain were assessed using study‐specific self‐report items. Adolescents were first asked whether they had experienced pain during the preceding 3 months; those responding affirmatively reported pain duration in months and indicated pain location, with multiple body sites selectable. Pain frequency, duration of pain episodes and pain duration were assessed using 5‐point Likert scales, whilst mean pain intensity over the previous 3 months was reported using the NRS. These variables were analysed descriptively and at the item level rather than as a composite scale. As these items were developed for descriptive epidemiological purposes, no internal consistency indices, composite score ranges or clinical cutoff values were applicable.

#### 2.3.3. Prevalence of Other Somatic Symptoms

The presence of additional somatic symptoms was assessed using a symptom checklist comprising common physical symptoms frequently associated with chronic pain conditions from the translated and linguistically validated German Paediatric Pain Questionnaire [19]. Adolescents were asked to indicate whether they had experienced specific symptoms, including queasiness, nausea, dizziness, sensitivity to light or sound, paleness, flushing, visual disturbances, smell or taste disturbances, unusual sensations in the hands, concentration difficulties and a general feeling of being unwell. Multiple responses were allowed in order to capture multisymptom presentations.

Items were analysed individually, and no composite score was calculated. As the checklist was developed for descriptive and exploratory purposes, it did not include subscales, a defined score range or established clinical cutoff values. Internal consistency indices (Cronbach′s *α*) were therefore not applicable. The checklist was not previously validated and was developed specifically for this study to provide a comprehensive overview of co‐occurring somatic symptoms in adolescents with chronic pain.

#### 2.3.4. Quality of Life, School Absence and Sleeping Difficulties

Pain‐related disability and HRQOL were assessed using the Hungarian version of the Pediatric Quality of Life Inventory 4.0 (PedsQL 4.0) Generic Core Scales [20–22]. The PedsQL is a 23‐item instrument comprising four subscales: physical functioning (eight items), emotional functioning (five items), social functioning (five items) and school functioning (five items). Items are rated on a 5‐point Likert scale, reverse‐scored and linearly transformed to a 0–100 scale, with higher scores indicating better HRQOL; a total scale score is calculated as the mean of all items. The instrument has demonstrated good to excellent internal consistency in its original validation (Cronbach′s *α* ranging from 0.68 to 0.90). The Hungarian version has been formally validated, and normative population data are available [23].

In accordance with Hungarian population‐based studies, extremely low, clinically pathological HRQOL was defined as a total scale score below 56.5 (mean −2 SD). Physical health was reflected by the physical functioning subscale, whilst psychosocial health was captured by the emotional, social and school functioning subscales. Additional indicators of functional disability included self‐reported school absenteeism (number of missed school days in the preceding 3 months) and sleeping difficulties, assessed with a binary (yes/no) question [5]. These items were analysed descriptively and were used as indicators of pain‐related functional impairment. As single‐item measures, internal consistency indices were not applicable.

#### 2.3.5. Pain‐Related Consequences: Healthcare Utilisation and Analgesic Usage

Pain‐related healthcare utilisation and analgesic usage were assessed using study‐specific single‐item self‐report questions as indicators of health‐related consequences. Participants were asked to report the number of pain‐related physician consultations and hospitalisations. Analgesic usage during the preceding 3 months was assessed using a 5‐point Likert scale: 1 (*monthly or less often*), 2 (*several times a month*), 3 (*once a week*), 4 (*several times a week*) and 5 (*daily*), with higher scores indicating more frequent medication use.

Items were analysed descriptively and at the item level, and no composite score was calculated. As these measures were used as surrogate indicators of pain‐related burden rather than as psychometric scales, they did not include subscales, defined score ranges or established clinical cutoff values. Internal consistency indices (Cronbach′s *α*) were therefore not applicable. This approach was consistent with previous paediatric chronic pain research [5].

We have summarised the utilised specific instruments according to the PedIMMPACT recommendations (Table 1) [15].

**Table 1:** PedIMMPACT domains and the specific instruments used.

Domain	Specific instrument
Pain intensity	NRS
Global judgement of satisfaction with treatment	Not applicable
Symptoms and adverse events	Other somatic symptoms questions
Physical functioning	PedsQL
Emotional functioning	PedsQL
Role functioning	PedsQL
Sleep	Sleeping difficulties questions
Economic factors	Healthcare utilisation and analgesic usage questions

### 2.4. Data Analysis

We deployed descriptive statistics for frequency counts to explore the prevalence of school stressors, harassments, pain characteristics and analgesic usage. We used the *χ* ^2^ test and Fisher test to evaluate differences in the prevalence of school stressors, harassments and analgesic usage between various study groups and categorical variables. We utilised the Mann–Whitney *U* test to evaluate differences between study groups for continuous variables (HRQOL scores, school absences and healthcare utilisation). We calculated odds ratios to assess the strength of the relationships between dependent (e.g. harassments) and independent (presence or absence of FAPDs) variables. A multivariate regression model was used to predict participants with extremely low quality of life within the FAPD group. The variables included the continuous variables that were hypothesised to play a role in the functional outcome of children with FAPD (HRQOL [under −2 SD], age, number of pain problems, pain intensity, duration of pain, frequency of pain, duration of pain attacks, number of other somatic symptoms, accusation, mobbing, verbal and physical harassment). Multicollinearity amongst the independent variables was assessed using the variance inflation factor (VIF). VIF values ranged between 1.01 and 1.08, indicating that multicollinearity was not a concern. The analyses were hypothesis‐driven, and the number of comparisons was limited; a strict Bonferroni correction was not applied.

Missing data were excluded from the analyses. Data were analysed using SPSS software Version 26.0 (IBM SPSS Statistics, IBM Corporation, Chicago, Illinois), and *p* < 0.05 was considered statistically significant.

## 3. Results

### 3.1. School Stressors and Frequent Harassment Were More Frequent Amongst Adolescents With FAPDs

**Figure 1:**
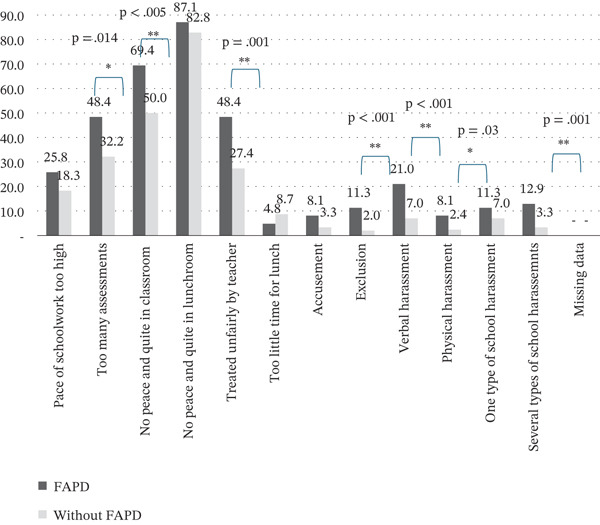
Comparison of frequency of school stressors and frequent (daily/weekly) harassment between FAPD and without FAPD groups ( ^∗^ *p* ≤ 0.05;  ^∗∗^ *p* ≤ 0.005).

Significantly, more adolescents with FAPDs reported that they experienced specific school stressors (too many assessments, no peace and quiet in the classroom and being treated unfairly by the teacher) compared to adolescents without FAPDs. The frequent (daily/weekly) harassment, as measured by exclusion, verbal and physical aggression and at least two types of harassment, was also significantly higher in the FAPD group (Figure 1).

Exclusion was 6.3 times more likely (OR 6.3; 95% CI 2.3–17.7) in the FAPD group (*p* < 0.001). Verbal aggression was also found to be significantly more frequent (OR 3.5; 95% CI 1.7–7.1; *p* < 0.001). Physical aggression was 3.5 times more likely (OR 3.5; 95% CI 1.2–10.5) to be significantly associated with functional abdominal pain (*p* = 0.03). It is noteworthy that 12.9% of adolescents with FAPD were exposed to more than one type of harassment (Figure 1).

### 3.2. High Prevalence of Extraintestinal and Multisite Pain and Higher Number of Other Somatic Symptoms Amongst Adolescents With FAPDs

54.8% of adolescents with a diagnosis of FAPD reported extraintestinal pain (pain outside of the abdomen) as their primary pain site. The most frequently reported extraintestinal pain was headache (19.4%).

Furthermore, 80.6% of adolescents identified two or more pain sites (multisite pain). A significant proportion of adolescents diagnosed with FAPDs (54.8%) suffered from chronic symptoms lasting for more than 1 year. In addition, 11.3% of adolescents with FAPDs reported that they experience pain more debilitatingly: on a continuous or daily basis. As much as 41.9% of the sample suffered from pain attacks lasting either a whole day or most of the day. These data may suggest that a significant proportion of adolescents with FAPDs experience long‐lasting, severe, daily pain (Table 2).

**Table 2:** Pain characteristics of the FAPD group (*N* [%] and medians and interquartile ranges [IQRs] are shown).

Measurement						
Primary pain site	Stomach	Head	Joints	Back	Other	Missing
*N* (%)	28 (45.2)	12 (19.4)	8 (12.9)	6 (9.7)	8 (12.8)	0 (0)
Pain duration	≤ 1 month	2 months	3 months	3–11 months	≥ 12 months	Missing
*N* (%)	5 (8.1)	4 (6.5)	10 (16.1)	6 (9.7)	34 (54.8)	3 (4.8)
Frequency of pain	1–3/month	1/week	Several/week	Daily	Continuous	Missing
*N* (%)	24 (38.7)	14 (22.6)	15 (24.2)	5 (8.1)	2 (3.2)	2 (3.2)
Mean pain intensity	NRS					Missing
Pain intensity median (IQR)	6.5 (5.0–8.0)					4 (6.5)
Duration of pain attacks	≤ 1 h	1–2 h	3–4 h	Most of the day	Whole day	Missing
*N* (%)	8 (12.9)	14 (22.6)	10 (16.1)	18 (29.0)	8 (12.9)	3 (4.8)

Other somatic symptoms were also significantly more prevalent in the FAPD group (Mann–Whitney *U* = 5494.5, *p* < 0.001).

Adolescents with FAPDs reported significantly poorer HRQOL and higher school absenteeism; 14.5% exhibited extremely low HRQOL.

**Figure 2:**
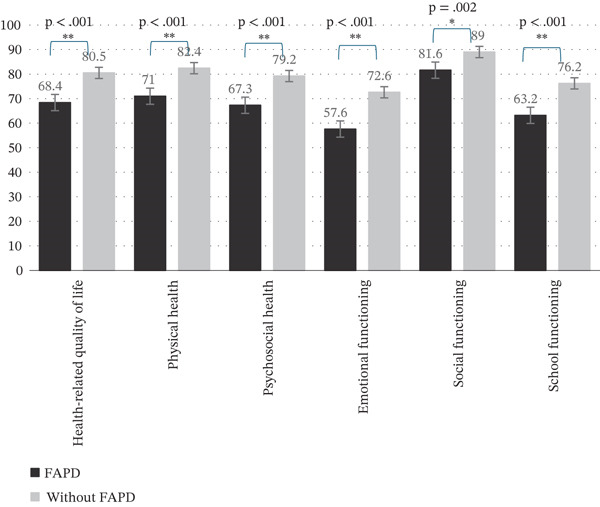
Health‐related quality of life of FAPD and without FAPD groups (Mann–Whitney *U* test ( ^∗^ *p* = 0.002;  ^∗∗^ *p* < 0.001).

We have found that adolescents with FAPDs reported statistically significantly poorer overall HRQOL (Figure 2). Furthermore, adolescents with FAPDs scored significantly lower on all subscales of the HRQOL questionnaire and exhibited poorer physical as well as psychosocial health. We also found that 14.5% of the adolescents with FAPDs exhibited extremely low (under −2 SD) HRQOL (defined as a total scale score lower than 56.5). They were 7.7 times more likely to have an extremely low HRQOL than adolescents without FAPDs (CI 2.9–19.9, *p* < 0.001).

Furthermore, adolescents with FAPDs reported significantly higher mean number of missed school days versus without FAPDs: median 8.5 (IQR 6.0–13.0) days versus 6.0 (IQR 2.0–10.0) days (Mann–Whitney *U* = 17,144.5, *p* < 0.001).

There was no statistically significant difference—albeit being almost two times higher—in the prevalence of sleeping difficulties amongst the FAPD group than in the group without FAPD.

### 3.3. Healthcare Utilisation and Analgesic Usage Are Higher in the FAPD Group

Although the differences were not statistically significant, the FAPD group consequently reported a higher use of the healthcare services: Both medical examination in the last 3 months (FAPD group: median 1.5 [IQR 1.0–9.3]; without FAPD: 1.0 [IQR 0.0–4.0]) and number of hospitalisation (FAPD group: median 1.5 [IQR 1.0–3.8]; without FAPD: 1.0 [IQR 1.0–2.0]) were higher.

**Figure 3:**
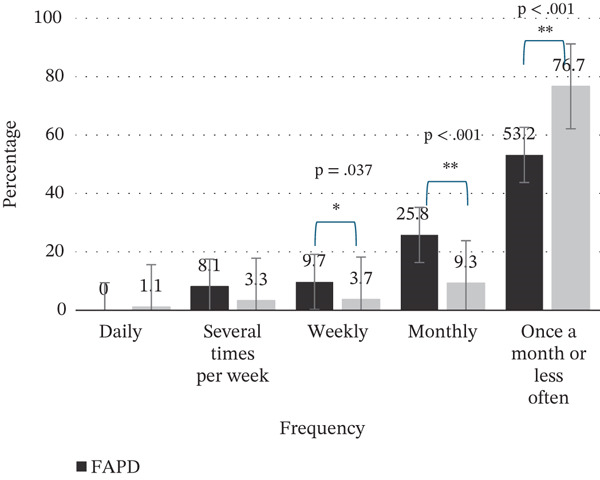
The analgesic usage of FAPD and without FAPD groups. Regular usage of analgesics is more frequent in the FAPD group ( ^∗^ *p* ≤ 0.05;  ^∗∗^ *p* < 0.001).

The previous trend was confirmed by the fact that analgesic usage was significantly higher in adolescents with FAPDs (overall analgesic usage FAPD median = 2.5 [IQR 1.0–4.0], without FAPD median = 1.0 [IQR 1.0–2.0]; *U* = 16,346.0; *p* < 0.001). The FAPD group reported a significantly higher usage of analgesics on a weekly (9.7% vs. 3.7%) and monthly (25.8% vs. 9.3%) basis versus the group without FAPD, suggesting that regular medication intake is more typical for this group (Figure 3).

### 3.4. Number of Pain Problems, Frequency of Pain and Verbal Harassment Are Associated With Extremely Low HRQOL

In multivariate linear regression analysis, we evaluated the relationship between extremely low HRQOL (under −2 SD) and age, number of pain problems, pain intensity, duration of pain, frequency of pain, duration of pain attacks, number of other somatic symptoms, accusation, mobbing, verbal and physical harassment within the FAPD group. Results showed that the number of pain problems, frequency of pain and verbal harassment were associated with extremely low quality of life (Table 3).

**Table 3:** Multivariate linear regression model (stepwise method) predicting extremely low HRQOL in the FAPD group.

Factors	*B* (95% CI)	Beta	*p* value	VIF
Constant	92.8		0.000	
Number of pain problems	−4.2	−0.3	0.025	1.07
Frequency of pain	−4.0	−0.3	0.007	1.08
Verbal harassment	−4.1	−0.4	0.000	1.01

*Note:* *R* ^2^ = 0.47.

Abbreviation: VIF, variance inflation factor.

## 4. Discussion

In this study, we used a pain therapeutic approach and the PedIMMPACT recommendations to explore the school‐related antecedents, pain characteristics and consequences of FAPDs in a cohort of Hungarian adolescents and to identify associated factors of poor HRQOL of adolescents.

Previous studies have hypothesised that school stressors and peer harassment represent psychosocial risk factors for the development of FAPDs. However, only a limited number of studies have examined this relationship, and amongst these, only two assessed FAPD prevalence using Rome criteria, whilst school‐related factors were largely restricted to punishment [8, 9]. By assessing multiple dimensions of school stress, the present study expands current knowledge on school‐related risk factors of FAPDs.

More than half of adolescents with FAPDs in our sample reported extraintestinal pain as the primary pain site, and multisite pain was present in 80.6% of cases. A substantial proportion also reported long‐lasting or frequent pain exceeding Rome cutoff thresholds. These findings are consistent with an emerging body of evidence showing that chronic abdominal pain frequently co‐occurs with pain in other body regions [8, 13, 24]. However, the relationship between extraintestinal pain, FAPDs and abdominal pain severity remains underinvestigated. A study from Sri Lanka assessed the severity of functional abdominal pain, and a recently published article by Chumpitazi et al. focused on this problem previously [8, 13, 24].

Multisite pain may be a possible indicator of a more severe disease course, as a previous clinical study suggested that children referred to inpatient treatment reported more pain locations [25].

The significantly higher prevalence of additional somatic symptoms amongst adolescents with FAPDs supports previous findings linking the disorder to elevated emotional burden and symptom severity, which may contribute to reduced HRQOL [26–28].

Consistent with earlier research, adolescents with FAPDs reported significantly lower HRQOL and more missed school days [4, 9, 29–34]. Importantly, our identification of a subgroup with clinically pathological HRQOL scores extends previous findings by demonstrating that severe functional impairment may affect a substantial subset of adolescents.

In contrast to some earlier studies, we did not find a significantly higher prevalence of sleep difficulties in adolescents with FAPDs. However, other studies reported increased sleep problems amongst adolescents with IBS and chronic pain [6, 35]. This may potentially reflect differences between FAPD subtypes [6].

Our results also parallel those of a Sri Lankan study, which reported higher rates of healthcare‐seeking behavior among adolescents with FAPDs, although the difference did not reach statistical significance [36]. In contrast, the significantly higher use of on‐demand pain medication (excluding psychiatric and neuropathic medications) is consistent with prior research [4, 27].

Our results suggest that adolescents with FAPDs seem to have not only pain complaints but may also have a widespread symptomatology such as additional somatic symptoms, psychosocial risk factors (like school stressors) and poorer quality of life.

The findings of the present study demonstrate that, beyond pain frequency, multisite pain and verbal aggression, both of which are frequently experienced by patients, may be significant predictors of extremely low quality of life. As HRQOL is a key indicator of retained functionality in chronic pain, these findings illustrate the interaction of biological, psychological and social factors in severe paediatric pain [11].

### 4.1. Clinical Implications

Within the healthcare and school system, our findings indicate that a subgroup of adolescents with FAPDs potentially experience chronic disabling pain, characterised by extremely low HRQOL and high functional burden. These adolescents are likely to have substantial limitations in daily functioning and increased healthcare needs, consistent with previous long‐term outcome studies [3].

Routine clinical care for adolescents with FAPDs might therefore extend beyond gastrointestinal symptoms and include screening for HRQOL, school stressors and multisite pain, even in paediatric gastroenterology outpatient settings [12, 37].

### 4.2. Research Implications

From a research perspective, our findings emphasise the importance of using PedIMMPACT‐aligned outcome domains, particularly HRQOL, to identify adolescents with clinically meaningful disability. The role of school stressors—especially verbal aggression—should be examined further in longitudinal studies to determine causal relationships and generalisability beyond Hungary.

The present study has several strengths. This is the first survey that uses detailed questionnaires to assess a comprehensive set of antecedents and outcome variables such as school stressors, pain characteristics, HRQOL, school absenteeism, healthcare utilisation and pain medication use to assess functional disability due to chronic pain amongst adolescents with FAPDs. These measurements were selected according to the PedIMMPACT recommendations and assessed seven of the eight recommended domains [15]. In addition, our study deployed a representative sample which allows for a more accurate identification of a cohort of patients with potential need for treatment. Finally, this is the first study to identify a subgroup of adolescents with FAPD patients with extremely low HRQOL and elucidate its predictors.

Our study has several limitations. The cross‐sectional setting does not allow for cause‐and‐effect conclusions. Nonetheless, the self‐report questionnaires of younger children could be inaccurate in some cases. Despite achieving a commendable response rate of 80.2%, approximately one‐fifth of the adolescents did not return the questionnaire. This could influence the results, as potential participants with health problems are more likely to respond.

A further limitation of the present statistical analysis is the absence of adjusted estimates, controlled multivariable models and model‐based count analyses. Subsequent investigations employing larger samples should utilise multivariable models to account for confounding factors such as age, sex and other relevant covariates.

The study was conducted in accordance with the Rome III criteria; the most recent Rome IV criteria were not utilised. However, it is noteworthy that the data obtained are comparable to those recently published in a European study using the Rome IV criteria [38]. Conversely, epidemiological studies conducted in Latin America have suggested a lower overall prevalence of FAPDs [28]. This observation did not replicate in a Turkish study, wherein the Rome IV criteria did not result in an alteration in the number of patients diagnosed with FAPDs according to the Rome III criteria [26].

FAPD diagnosis relies exclusively on QPGS‐III self‐report without clinical examination. This increases misclassification risk (e.g. organic pathology). Furthermore, the lack of clinician‐administered assessments may have reduced our ability to differentiate between functional and organic causes of abdominal pain. The interpretation of our findings is further limited by the lack of data on psychological comorbidities, including attention‐deficit/hyperactivity disorder and autism spectrum disorders. Consequently, the stress factor scores of participants with such comorbidity may vary considerably.

A limitation of this study is that data were collected in 2016 and are reported retrospectively. Whilst contextual changes in educational and social environments may have occurred, the fundamental associations between chronic pain, functional impairment and school stress are unlikely to have substantially changed; nevertheless, future studies using more recent data are needed to evaluate potential temporal trends. Importantly, due to the lack of comparable Hungarian data in this field, we believe that reporting these findings remains relevant and contributes valuable population‐based evidence.

## 5. Conclusions

The results from the present study highlight the importance of improved pain assessment approaches for adolescents with FAPDs. Failing this, the frequently quoted prediction published by John Apley in the foreword of his book remains true: ‘Little belly‐achers grow up to be big “belly‐achers”– and they will “belly‐ache” about more than the abdomen’ [39].

**Nomenclature**
FAPD: functional abdominal pain disorder
FAPS: functional abdominal pain syndrome
HRQOL: health‐related quality of life
IBS: irritable bowel syndrome
IQR: interquartile range
NRS: numeric rating scale
PedIMMPACT: Core Outcome Domains and Measures for Pediatric Acute and Chronic/Recurrent Pain Clinical Trials Recommendations
PedsQL: Pediatric Quality of Life Inventory
QPGS‐III: Questionnaire for Paediatric Gastrointestinal Symptoms Rome *ΙΙΙ* Edition
SD: standard deviation
VIF: variance inflation factor

## Author Contributions

J.M. coordinated the study, built the database, analysed the data and wrote the manuscript. A.V. analysed the data, wrote the Results section and prepared the tables. S.Á. analysed the data, wrote sections of the manuscript (Introduction and Discussion) and revised the manuscript.

## Funding

This study has been funded by the Semmelweis University Doctoral School.

## Disclosure

The funding body played no role in the design of the study and the collection, analysis and interpretation of data and in writing the manuscript.

## Ethics Statement

Ethical approval for this study was granted by the Hungarian National Ethical Committee on 16 December 2015 (Protocol Number 59678–1/2015/EKU). Written informed consent was obtained from all participating adolescents and from their parents or legal guardians prior to participation.

## Consent

The authors have nothing to report.

## Conflicts of Interest

The authors declare no conflicts of interest.

## Data Availability

The data that support the findings of this study are available from the corresponding author upon reasonable request.

## References

[1] Korterink J. J. , Diederen K. , Benninga M. A. , and Tabbers M. M. , Epidemiology of Pediatric Functional Abdominal Pain Disorders: A Meta-Analysis, PLoS One. (2015) 10, e0126982, 10.1371/journal.pone.0126982, 2-s2.0-84930645334. 25992621 PMC4439136

[2] Hart S. L. , Somatic Symptoms, Peer and School Stress, and Family and Community Violence Exposura Among Urban Elementary School Children, Journal of Behavioral Medicine. (2013) 18, 1199–1216. 10.1007/s10865-012-9440-2 PMC3726557 22772584

[3] Hotopf M. , Carr S. , Mayou R. , Wadsworth M. , Wessely S. , Hotopf M. , Carr S. , Mayou R. , and Michael Wadsworth S. W. , Why Do Children Have Chronic Abdominal Pain, and What Happens to Them When They Grow Up? Population Based Cohort Study, BMJ. (1998) 316, no. 7139, 1196–1200, 10.1136/bmj.316.7139.1196, 2-s2.0-0032542957. 9552994 PMC28520

[4] Saps M. , Seshadri R. , Sztainberg M. , Schaffer G. , Marshall B. M. , and Di Lorenzo C. , A Prospective School-Based Study of Abdominal Pain and Other Common Somatic Complaints in Children, Journal of Pediatrics. (2009) 154, no. 3, 322–326, 10.1016/j.jpeds.2008.09.047, 2-s2.0-60249094443, 19038403. 19038403

[5] Zernikow B. , Wager J. , Hechler T. , Hasan C. , Rohr U. , Dobe M. , Meyer A. , Hübner-Möhler B. , Wamsler C. , and Blankenburg M. , Characteristics of Highly Impaired Children With Severe Chronic Pain: A 5-Year Retrospective Study on 2249 Pediatric Pain Patients, Bmc Pediatrics. (2012) 12, 10.1186/1471-2431-12-54, 2-s2.0-84860996710. PMC3404028 22591492

[6] Zhou H.-Q. , Yao M. , Chen G.-Y. , Ding X.-D. , Chen Y.-P. , and Li D.-G. , Functional Gastrointestinal Disorders Among Adolescents With Poor Sleep: A School-Based Study in Shanghai, Sleep and Breathing. (2012) 16, 1211–1218, 10.1007/s11325-011-0635-5, 2-s2.0-84877112093. 22203339

[7] Hjern A. , Alfven G. , and Östberg V. , School Stressors, Psychological Complaints and Psychosomatic Pain, Acta Paediatrica. (2008) 97, no. 1, 112–117, 10.1111/j.1651-2227.2007.00585.x, 2-s2.0-38349052277, 18076714. 18076714

[8] Devanarayana N. M. , Mettananda S. , Liyanarachchi C. , Nanayakkara N. , Mendis N. , Perera N. , and Rajindrajith S. , Abdominal Pain-Predominant Functional Gastrointestinal Diseases in Children and Adolescents: Prevalence, Symptomatology, and Association With Emotional Stress, Journal of Pediatric Gastroenterology And Nutrition. (2011) 53, no. 6, 659–665, 10.1097/MPG.0b013e3182296033, 2-s2.0-82355173470, 21697745. 21697745

[9] Udoh E. , Devanarayana N. M. , Rajindrajith S. , Meremikwu M. , and Benninga M. A. , Abdominal Pain-Predominant Functional Gastrointestinal Disorders in Adolescent Nigerians, Journal of Pediatric Gastroenterology and Nutrition. (2016) 62, 588–593, 10.1097/MPG.0000000000000994, 2-s2.0-84944339893. 26465793

[10] Könning A. , Rosenthal N. , Brown D. , Stahlschmidt L. , and Wager J. , Severity of Chronic Pain in German Adolescent School Students-A Cross-Sectional Study, Clinical Journal of Pain. (2021) 37, no. 2, 118–125, 10.1097/AJP.0000000000000898, 33165023. 33165023

[11] Claus B. B. , Stahlschmidt L. , Dunford E. , Major J. , Harbeck-Weber C. , Bhandari R. P. , Baerveldt A. , Neb V. , Grochowska K. , Hübner-Möhler B. , Zernikow B. , and Wager J. , Intensive Interdisciplinary Pain Treatment for Children and Adolescents With Chronic Noncancer Pain: A Preregistered Systematic Review and Individual Patient Data Meta-Analysis, Pain. (2022) 163, 2281–2301, 10.1097/j.pain.0000000000002636. 35297804

[12] Chu A. S. , Torres L. , Kao G. , Gilbert C. , Monico E. C. , and Chumpitazi B. P. , Multidisciplinary Care for Refractory Pediatric Functional Abdominal Pain Decreases Emergency and Inpatient Utilization, Journal of Pediatric Gastroenterology and Nutrition. (2022) 74, no. 2, 248–252, 10.1097/MPG.0000000000003305, 34560729. 34560729 PMC8799479

[13] Chumpitazi B. P. , Palermo T. M. , Hollier J. M. , Self M. M. , Czyzewski D. , Weidler E. M. , Heitkemper M. , and Shulman R. J. , Multisite Pain Is Highly Prevalent in Children With Functional Abdominal Pain Disorders and Is Associated With Increased Morbidity, Journal of Pediatrics. (2021) 236, 131–136, 10.1016/j.jpeds.2021.04.059, 33940018. 33940018 PMC8403143

[14] Shelby G. D. , Shirkey K. C. , Sherman A. L. , Beck J. E. , Haman K. , Shears A. R. , Horst S. N. , Smith C. A. , Garber J. , and Walker L. S. , Functional Abdominal Pain in Childhood and Long-Term Vulnerability to Anxiety Disorders, Pediatrics. (2013) 132, 475–482, 10.1542/peds.2012-2191, 2-s2.0-84884585182. 23940244 PMC3876748

[15] McGrath P. J. , Walco G. A. , Turk D. C. , Dworkin R. H. , Brown M. T. , Davidson K. , Eccleston C. , Finley G. A. , Goldschneider K. , Haverkos L. , Hertz S. H. , Ljungman G. , Palermo T. , Rappaport B. A. , Rhodes T. , Schechter N. , Scott J. , Sethna N. , Svensson O. K. , Stinson J. , von Baeyer C. L. , Walker L. , Weisman S. , White R. E. , Zajicek A. , and Zeltzer L. , Core Outcome Domains and Measures for Pediatric Acute and Chronic/Recurrent Pain Clinical Trials: PedIMMPACT Recommendations, Journal of Pain. (2008) 9, 771–783, 10.1016/j.jpain.2008.04.007, 2-s2.0-49649128100. 18562251

[16] Major J. , Ádám S. , Major J. , and Ádám S. , Self-Reported Specific Learning Disorders and Risk Factors Among Hungarian Adolescents With Functional Abdominal Pain Disorders: A Cross Sectional Study, BMC pediatrics. (2020) 20, 10.1186/s12887-020-02167-w. PMC7275532 32505201

[17] Roman-Juan J. , Mychasiuk R. , Macfarlane G. J. , Hood A. M. , and Noel M. , National Income Inequality and Adolescent Chronic Pain: A Time Series Analysis of 29 Countries, Pain. (2026) 167, no. 1, 102–109, 10.1097/J.PAIN.0000000000003756. 40844490

[18] Birnie K. A. , Hundert A. S. , Lalloo C. , Nguyen C. , and Stinson J. N. , Recommendations for Selection of Self-Report Pain Intensity Measures in Children and adolescents: a systematic review and quality assessment of measurement properties, Pain. (2019) 160, no. 1, 5–18, 10.1097/j.pain.0000000000001377, 2-s2.0-85059194470, 30180088. 30180088

[19] Schroeder S. , Hechler T. , Denecke H. , Müller-Busch M. , Martin A. , Menke A. , and Zernikow B. , Deutscher Schmerzfragebogen für Kinder, Jugendliche und deren Eltern (DSF-KJ), Der Schmerz. (2010) 24, 23–37, 10.1007/s00482-009-0864-8, 2-s2.0-77649232558. 20108103

[20] Varni J. , Seid M. , and Kurtin P. , The PedsQL™ 4.0 as a Pediatric Population Health Measure: Feasibility, Reliability, and Validity, Ambulatory Pediatrics. (2003) 3, no. 6, 329–341, 10.1367/1539-4409(2003)003<0329:TPAAPP>2.0.CO;2, 14616041. 14616041

[21] Varni J. W. , Seid M. , and Kurtin P. S. , PedsQL 4.0: Reliability and Validity of the Pediatric Quality of Life Inventory Version 4.0 Generic Core Scales in Healthy and Patient Populations, Medical Care. (2001) 39, 800–812, 10.1097/00005650-200108000-00006, 2-s2.0-0035432179. 11468499

[22] Varni J. W. , Seid M. , and Rode C. A. , The PedsQL: Measurement Model for the Pediatric Quality of Life Inventory, Medical Care. (1999) 37, 126–139, 10.1097/00005650-199902000-00003, 2-s2.0-0033073557. 10024117

[23] Berkes A. , Riszter M. , Felszeghy E. , Pataki I. , and Mogyorósy G. , Measurement Properties of the Hungarian Version of the Pediatric Quality of Life Inventory 4.0: Health Related Quality of Life and Associated Characteristics of the School Children in Hungary, Applied Research in Quality of Life. (2019) 14, no. 4, 981–1000, 10.1007/s11482-018-9639-7, 2-s2.0-85046036749.

[24] Chitkara D. K. , Rawat D. J. , and Talley N. J. , The Epidemiology of Childhood Recurrent Abdominal Pain in Western Countries: A Systematic Review, Official journal of the American College of Gastroenterology| ACG. (2005) 100, no. 8, 1868–1875, 10.1111/j.1572-0241.2005.41893.x, 2-s2.0-25644436397, 16086724. 16086724

[25] Hechler T. , Martin A. , Blankenburg M. , Schroeder S. , Kosfelder J. , Hölscher L. , Denecke H. , and Zernikow B. , Specialized Multimodal Outpatient Treatment for Children With Chronic Pain: Treatment Pathways and Long-Term Outcome, European Journal of Pain. (2011) 15, no. 9, 976–984, 10.1016/j.ejpain.2011.03.001, 2-s2.0-80053568558, 21440471. 21440471

[26] Demirören K. , Göney B. , Bostanc M. , and Ekici D. , A Comparison Between Rome III and Rome IV Criteria in Children With Chronic Abdominal Pain: A Prospective Observational Cohort Study, Turkish Journal of Gastroenterology. (2022) 33, no. 11, 979–984, 10.5152/tjg.2022.21893, 35946891. PMC9797798 35946891

[27] Roth-Isigkeit A. , Thyen U. , Stö H. , Schwarzenberger J. , and Schmucker P. , Pain Among Children and Adolescents: Restrictions in Daily Living and Triggering Factors, Pediatrics. (2005) 115, no. 2, e152–e162, 10.1542/peds.2004-0682, 2-s2.0-17844401744, 15687423. 15687423

[28] Saps M. , Velasco-Benitez C. A. , Langshaw A. H. , and Ramírez-Hernández C. R. , Prevalence of Functional Gastrointestinal Disorders in Children and Adolescents: Comparison Between Rome III and Rome IV Criteria, Journal of Pediatrics. (2018) 199, 212–216, 10.1016/j.jpeds.2018.03.037, 2-s2.0-85046704369. 29747935

[29] Aziz I. , Palsson O. S. , Törnblom H. , Sperber A. D. , Whitehead W. E. , and Simrén M. , The Prevalence and Impact of Overlapping Rome IV-Diagnosed Functional Gastrointestinal Disorders on Somatization, Quality of Life, and Healthcare Utilization: A Cross-Sectional General Population Study in Three Countries, American Journal of Gastroenterology. (2018) 113, no. 1, 86–96, 10.1038/ajg.2017.421, 2-s2.0-85042871346, 29134969. 29134969

[30] Ranasinghe N. , Devanarayana N. M. , Rajindrajith S. , Perera M. S. , Nishanthinie S. , Warnakulasuriya T. , and de Zoysa P. T. , Functional Gastrointestinal Diseases and Psychological Maladjustment, Personality Traits and Quality of Life, BMC gastroenterology. (2018) 18, no. 1, 10.1186/s12876-018-0760-8, 2-s2.0-85042561587, 29486708. PMC5830068 29486708

[31] Rutten J. M. T. M. , Benninga M. A. , and Vlieger A. M. , IBS and FAPS in Children: A Comparison of Psychological and Clinical Characteristics, Journal of Pediatric Gastroenterology and Nutrition. (2014) 59, no. 4, 493–499, 10.1097/MPG.0000000000000452, 2-s2.0-84917736813, 24897168. 24897168

[32] Sagawa T. , Okamura S. , Kakizaki S. , Zhang Y. , Morita K. , and Mori M. , Functional Gastrointestinal Disorders in Adolescents and Quality of School Life, Journal of Gastroenterology And Hepatology. (2013) 28, no. 2, 285–290, 10.1111/j.1440-1746.2012.07257.x, 2-s2.0-84872820843. 22988951

[33] Warschburger P. , Hänig J. , Friedt M. , Posovszky C. , Schier M. , and Calvano C. , Health-Related Quality of Life in Children With Abdominal Pain due to Functional or Organic Gastrointestinal Disorders, Journal of Pediatric Psychology. (2014) 39, 45–54, 10.1093/jpepsy/jst070, 2-s2.0-84892720463. 24055816

[34] Zeevenhooven J. , Timp M. L. , Singendonk M. M. J. , Benninga M. A. , and Tabbers M. M. , Definitions of Pediatric Functional Abdominal Pain Disorders and Outcome Measures: A Systematic Review, Journal of Pediatrics. (2019) 212, 52–59.e16, 10.1016/j.jpeds.2019.04.048, 2-s2.0-85068129502. 31277898

[35] Kumagai H. , Yokoyama K. , Imagawa T. , and Yamagata T. , Functional Dyspepsia and Irritable Bowel Syndrome in Teenagers: Internet Survey, Pediatrics International. (2016) 58, no. 8, 714–720, 10.1111/ped.12884, 2-s2.0-84983335660, 26690554. 26690554

[36] Devanarayana N. M. , Rajindrajith S. , and Benninga M. A. , Quality of Life and Health Care Consultation in 13 to 18 Year Olds With Abdominal Pain Predominant Functional Gastrointestinal Diseases, BMC Gastroenterol. (2014) 14, 10.1186/1471-230X-14-150, 2-s2.0-84924262735. PMC4236590 25145589

[37] Wager J. , Ruhe A. , Stahlschmidt L. , Leitsch K. , Claus B. B. , Häuser W. , Brähler E. , Dinkel A. , Kocalevent R. , and Zernikow B. , Long-Term Outcomes of Children With Severe Chronic Pain: Comparison of Former Patients With a Community Sample, European Journal of Pain. (2021) 25, no. 6, 1329–1341, 10.1002/ejp.1754, 33619774. 33619774

[38] Strisciuglio C. , Cenni S. , Serra M. R. , Dolce P. , Kolacek S. , Sila S. , Trivic I. , Lev M. R. B. , Shamir R. , Kostovski A. , Papadopoulou A. , Roma E. , Katsagoni C. , Jojkic-Pavkov D. , Salvatore S. , Pensabene L. , Scarpato E. , Miele E. , and Staiano A. , Functional Gastrointestinal Disorders in Mediterranean Countries According to Rome IV Criteria, Journal of Pediatric Gastroenterology And Nutrition. (2022) 74, 361–367, 10.1097/mpg.0000000000003358. 35226645

[39] Apley J. , The Child With Abdominal Pains, 1975, 2nd edition, Blackwell Publishing Ltd.

